# Supplementary material for: Growth of electroautotrophic microorganisms using hydrovoltaic energy through natural water evaporation
Source: Nat Commun. 2024 Jun 11;15:4992. doi: 10.1038/s41467-024-49429-0 (PMC11166942; doi:10.1038/s41467-024-49429-0)
Supplement: Supplementary file 1 — Supplementary Information [file 41467_2024_49429_MOESM1_ESM.pdf]

Supplementary Information for

Growth of electroautotrophic microorganisms using hydrovoltaic energy through natural water evaporation

Guoping Ren[1], Jie Ye[1], Qichang Hu[1], Dong Zhang[1], Yong Yuan[2]* and Shungui Zhou[1]*

[1] Fujian Provincial Key Laboratory of Soil Environmental Health and Regulation, College of Resources and Environment, Fujian Agriculture and Forestry University; Fuzhou, 350002, China.

[2] Guangdong Key Laboratory of Environmental Catalysis and Health Risk Control, School of Environmental Science and Engineering, Institute of Environmental Health and Pollution Control, Guangdong University of Technology, Guangzhou 510006, China

*Corresponding authors. Email: yyuan2017@gdut.edu.cn (Yuan Y.); sgzhou@fafu.edu.cn (Zhou S.)

This PDF file includes:

Supplementary Tables 1 to 8

Supplementary Figures 1 to 12

Supplementary References

Supplementary Table 1. Energy efficiency of the ambient energy to electron energy conversion.

Evaporated quantity of water (ΔW_w, g)	Absorbed heat by water evaporation ($\Delta E_{w,}$ J)	Generated current (I, μA)	Generated volatge (V, V)	Generated power (P, μW)	Generated energy (ΔE_e, J)	Energy efficiency ($E_{w\rightarrow e}$, ‰)
13.71	30994.39	0.63	0.32	5.04×10^{-2}	0.22	7.10×10^{-3}

The energy conversion efficiency $E_{w\rightarrow e}$ (‰) of the absorbed heat energy by water evaporation to electricity energy was calculated as follows:

$$E_{w\rightarrow e} = \Delta E_e / \Delta E_w \times ‰ \quad (1)$$

Where ΔE_e (J) is the generated electron energy by water evaporation, ΔE_w (J) is the absorbed heat by water evaporation.

$$\Delta E_e = \int P\,dt = P \times 86400 \times 50 \times 10^{-6} \quad (2)$$

Where P (μW) is the generated maximum power by water evaporation ($P = I \times V / 4$)[1], I is generated maximum current during water evaporation, V is generated maximum voltage during water evaporation, factor 4 is the constant used to calculate the maximum power when the hydrovoltaic I-V curve approximates a straight line[2], the 86400 (s/day) is the number of seconds in a day, 50 (days) is the running time of the water evaporation, 10^{-6} is a microampere (μA) to amperage (A).

$$\Delta E_w = W_w \times 2260 \quad (3)$$

Where W_w is evaporated weight (g) of water during 50 days, 2,260 the enthalpy of water vaporization (J/g-water)[3].

Supplementary Table 2. Energy conversion efficiency of the hydrovoltaic electron energy to chemical energy in microorganisms.

Generated number of electrons (n_{e-}, μmol)	Nitrate reduction (n_{NR}, μmol)	Electrons required for nitrate reduction ($n_{e- \text{ for NR}}$, μmol)	Biomass increase ($W_{biomass}$, μg)	Electrons required for biomass increase ($n_{e- \text{ for biomass}}$, μmol)	Energy of cell proliferation (ΔE_b, μmol)	Energy efficiency ($E_{e\rightarrow b}$, %)
29.0	3.50	7.00	165.83	20.73	27.73	95.6

The energy conversion efficiency ($E_{e\rightarrow b}$) of the hydrovoltaic electron energy to chemical energy in microorganisms was calculated as follows:

$$E_{e\rightarrow b} = \Delta E_b \,/\, n_{e-} \quad (5)$$

$$\Delta E_b = n_{e- \text{ for NR}} + n_{e- \text{ for biomass}} \quad (4)$$

where ΔE_b is the energy required for microbial growth (including biomass increase and nitrate reduction), $n_{e- \text{ for NR}}$ is the electrons required for nitrate reduction, $n_{e- \text{ for biomass}}$ is the electrons required for biomass increase. the number (n_{e-}) of the generated electrons by water evaporation was calculated as follows[4]:

$$n_{e-} = \int I\, dt \,/F \quad (6)$$

where F is the Faraday constant (96485 C/mol).

$$n_{e- \text{ for NR}} = n_{NR} \times 2 \quad (7)$$

where n_{NR} (μmol) is the moles of NO_3^- reduction, 2 is the minimum number of electrons required for NO_3^- reduction[5].

$$n_{e- \text{ for biomass}} = W_{biomass} \,/\, 8 \quad (8)$$

where $W_{biomass}$ is the biomass increase (the data is based on COD concentrations before and after water evaporation during 50 days), 1 mol e^- eq = 8 g-COD[4,6].

Supplementary Table 3. Sterilized bacterial medium for *Rhodopseudomonas palustris* (g/L).

Components	Medium I<br>for bacterial culture	Medium II<br>for hydrovoltaic experiment
$CaCl_2$	0.1	0.1
KH_2PO_4	1.0	1.0
$NaHCO_3$	3.0	3.0
NH_4Cl	1.0	1.0
$MgCl_2$	0.5	0.5
NaCl	1.0	1.0
Ascorbic acid	0.3	0.3
KNO_3	0.5	0.04
Succinate	1.0	--
Acetate	1.0	--
Peptone	0.5	--
Yeast	0.5	--
Vitamin solution 1	1 mL	1 mL
Trace element solution 1	1 mL	1 mL

Vitamin solution 1 (g/L)

Components	Concentration
biotin	0.10
nicotinic acid	0.35
vitamin B1	0.30
4-aminobenzoic acid	0.20
pyridoxamine dihydrochloride	0.10
calcium pantothenate	0.10
vitamin B12	0.05

Trace element solution 1 (g/L)

Components	Concentration
$FeCl_2{\cdot}4H_2O$	1.80
$CoCl_2{\cdot}6H_2O$	0.25
$NiCl_2{\cdot}6H_2O$	0.01
$CuCl_2{\cdot}2H_2O$	0.01
$MnCl_2{\cdot}4H_2O$	0.70
$ZnCl_2$	0.10
H_3BO_3	0.50
$NaMnO_4{\cdot}2H_2O$	0.03
$NaSeO_3{\cdot}5H_2O$	0.01

Supplementary Table 4 Composition of media for *Geobacter sulfurreducens*.

	Medium I for bacterial culture	Medium II for hydrovoltaic experiment
$CaCl_2 \cdot 2H_2O$	0.04 g	0.04 g
$MgSO_4 \cdot 7H_2O$	0.1 g	0.1 g
$NaHCO_3$	1.8 g	1.8 g
$Na_2CO_3 \cdot H_2O$	0.5 g	0.5 g
1 mM Na_2SeO_4	1.0 mL	1.0 mL
Na Acetate·$3H_2O$	2.04 g	--
Fumaric Acid	4.64 g	--
NB Salts	10 mL	10 mL
Trace mineral solution 2	10 mL	10 mL
Vitamin solution 2	15 mL	15 mL

NB Salts		Vitamin solution 2		Trace mineral solution 2	
$KH_2PO_4 \cdot H_2O$	42 g/L	Biotin	0.002 g/L	NTA	2.14 g/L
$K_2HPO_4 \cdot 2H_2O$	22 g/L	Pantothenic Acid	0.005 g/L	$MnCl_2 \cdot 4H_2O$	0.1 g/L
NH_4Cl	20 g/L	B-12	0.0001 g/L	$FeSO_4 \cdot 7H_2O$	0.3 g/L
KCl	38 g/L	p-aminobenzoic Acid	0.005 g/L	$CoCl_2 \cdot 6H_2O$	0.17 g/L
		Thioctic Acid	0.005 g/L	$ZnSO_4 \cdot 7H_2O$	0.2 g/L
		Nicotinic Acid	0.005 g/L	$CuCl_2 \cdot 2H_2O$	0.03 g/L
		Thiamine	0.005 g/L	$AlK(SO_4)_2 \cdot 12H_2O$	0.005 g/L
		Riboflavin	0.005 g/L	H_3BO_3	0.005 g/L
		Pyridoxine HCl	0.01 g/L	$Na_2MoO_4 \cdot 2H_2O$	0.09 g/L
		Folic Acid	0.002 g/L	$NiSO_4 \cdot 6H_2O$	0.11 g/L
				$Na_2WO_4 \cdot 2H_2O$	0.02 g/L

Supplementary Table 5 Composition of media for *Shewanella oneidensis*.

	Medium I<br>for bacterial culture	Medium II<br>for hydrovoltaic experiment
NH_4Cl	--	0.46 g/L
K_2HPO_4	--	2.25 g/L
KH_2PO_4	--	2.25 g/L
$MgSO_4$ $7H_2O$	--	0.117 g/L
$(NH_4)_2SO_4$	--	0.225 g/L
Trace mineral solution 3	--	5 mL
Vitamin solution 3	--	5 mL
Cysteine	--	0.30 g/L
LB Broth	21.0 g/L	--

Vitamin solution 3		Trace mineral solution 3	
Biotin	2.0 mg/L	Nitrilotriacetic acid	1500.0 mg/L
Folic acid	2.0 mg/L	$MnCl_2$ $4H_2O$	100.0 mg/L
Pyridoxine HCl	20.0 mg/L	$FeSO_4$ $7H_2O$	300.0 mg/L
Thiamine	5.0 mg/L	$CoCl_2$ $6H_2O$	170.0 mg/L
Nicotinic acid	5.0 mg/L	$ZnCl_2$	100.0 mg/L
Pantothenic acid	5.0 mg/L	$CuSO_4$ $5H_2O$	40.0 mg/L
Vitamin B_{12}	0.1 mg/L	$AlK(SO_4)_2$ $12H_2O$	5.0 mg/L
P-aminobenzoic acid	5.0 mg/L	H_3BO_3	5.0 mg/L
Thioctic acid	5.0 mg/L	Na_2MoO_4	90.0 mg/L
		$NiCl_2$	120.0 mg/L
		$NaWO_4$ $2H_2O$	20.0 mg/L
		Na_2SeO_4	100.0 mg/L

Supplementary Table 6 Composition of media for *Escherichia coli and Bacillus subtilis*.

	Medium I for bacterial culture	Medium II for hydrovoltaic experiment
LB Broth	21.0 g/L	--
$CaCl_2 \cdot 2H_2O$	--	0.04 g
$MgSO_4 \cdot 7H_2O$	--	0.1 g
$NaHCO_3$	--	1.8 g
$Na_2CO_3 \cdot H_2O$	--	0.5 g
1 mM Na_2SeO_4	--	1.0 mL
NB Salts	--	10 mL
Trace mineral solution 2	--	10 mL
Vitamin solution 2	--	15 mL

Supplementary Table 7 Composition of media for *Moorella thermoacetica*.

	Medium I for bacterial culture	Medium II for hydrovoltaic experiment
NaCl	0.40 g/L	0.40 g/L
NH_4Cl	0.40 g/L	0.40 g/L
$MgSO_4 \cdot 7H_2O$	0.33 g/L	0.33 g/L
CaCl	0.05 g/L	0.05 g/L
KCl	0.25 g/L	0.25 g/L
K_2HPO_4	0.64 g/L	0.64 g/L
β-glycerophosphate·2Na·xH_2O	0.80 g/L	--
$NaHCO_3$	2.50 g/L	2.50 g/L
Vitamin solution 4	10 mL	10 mL
Trace mineral solution 4	10 mL	10 mL
Yeast Extract	0.50 g/L	--
Tryptone	0.50 g/L	--
Cysteine	0.30 g/L	0.30 g/L

Vitamin solution 4		Trace mineral solution 4	
Pyridoxine·HCl	10.0 mg/L	Nitriloacetic acid	2000.0 mg/L
Thiamine·HCl	5.0 mg/L	$MnSO_4 \cdot H_2O$	1000.0 mg/L
Riboflavin	5.0 mg/L	$Fe(SO_4)_2(NH_4)_2 \cdot 6H_2O$	800.0 mg/L
Nicotinic acid	5.0 mg/L	$CoCl_2 \cdot 6H_2O$	200.0 mg/L
Calcium D-(+)-pantothenate	5.0 mg/L	$ZnSO_4 \cdot 7H_2O$	0.2 mg/L
Thioctic acid	5.0 mg/L	$CuCl_2 \cdot 2H_2O$	20.0 mg/L
Biotin	2.0 mg/L	$NiCl_2 \cdot 6H_2O$	20.0 mg/L
Folic acid	2.0 mg/L	$Na_2MoO_4 \cdot 2H_2O$	20.0 mg/L
Vitamin B_{12}	0.1 mg/L	Na_2SeO_4	20.0 mg/L
		Na_2WO_4	20.0 mg/L

Supplementary Table 8 Composition of media for *Thiobacillus denitrificans*.

	Medium I for bacterial culture	Medium II for hydrovoltaic experiment
KH_2PO_4	2.0 g/L	2.0 g/L
KNO_3	2.0 g/L	--
NH_4Cl	1.0 g/L	--
$MgSO_4 \cdot 7H_2O$	0.8 g/L	0.8 g/L
$Na_2S_2O_3 \cdot 5H_2O$	5.0 g/L	--
$NaHCO_3$	1.0 g/L	1.0 g/L
$FeSO_4 \cdot 7H_2O$	2.0 mg/L	2.0 mg/L
H_2SO_4 (0.1 N)	1.0 mL	1.0 mL
Trace element solution 5-1	2.0 mL	2.0 mL
Trace element solution 5-2	2.0 mL	2.0 mL
Lactate	0.3 g/L	0.3 g/L

Trace element solution 5-1		Trace element solution 5-2	
EDTA	0.50 g/L	$ZnSO_4 \cdot 7H_2O$	0.10 g/L
$FeSO_4 \cdot 7H_2O$	0.20 g/L	$MnCl_2 \cdot 4H_2O$	0.03 g/L
Trace element solution 3	100.0 mL	H_3BO_3	0.30 g/L
		$CoCl_2 \cdot 6H_2O$	0.20 g/L
		$CuCl_2 \cdot 2H_2O$	0.01 g/L
		$NiCl_2 \cdot 6H_2O$	0.02 g/L
		$Na_2MoO_4 \cdot 2H_2O$	0.03 g/L

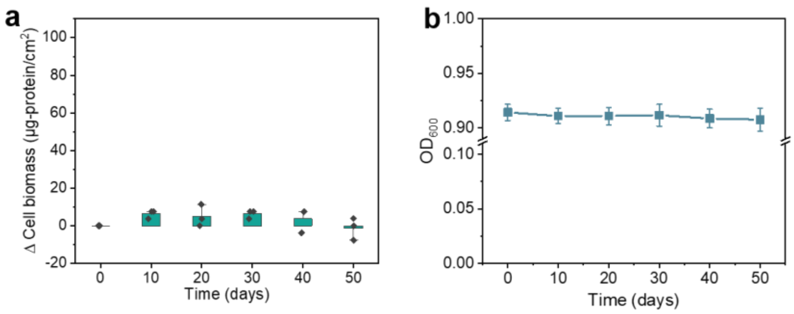

Supplementary Fig. 1 | Effect of ascorbic acid on the growth of *R. palustris*. a, OD_{600} of *R. palustris* incubated in the presence of ascorbic acid under a dark and anaerobic environment. **b**, Cell biomass (total protein) accumulation of *R. palustris* in the presence of ascorbic acid under a dark and anaerobic environment. Data represent mean ± SD from *n* = 3 technical replicates from one experiment.

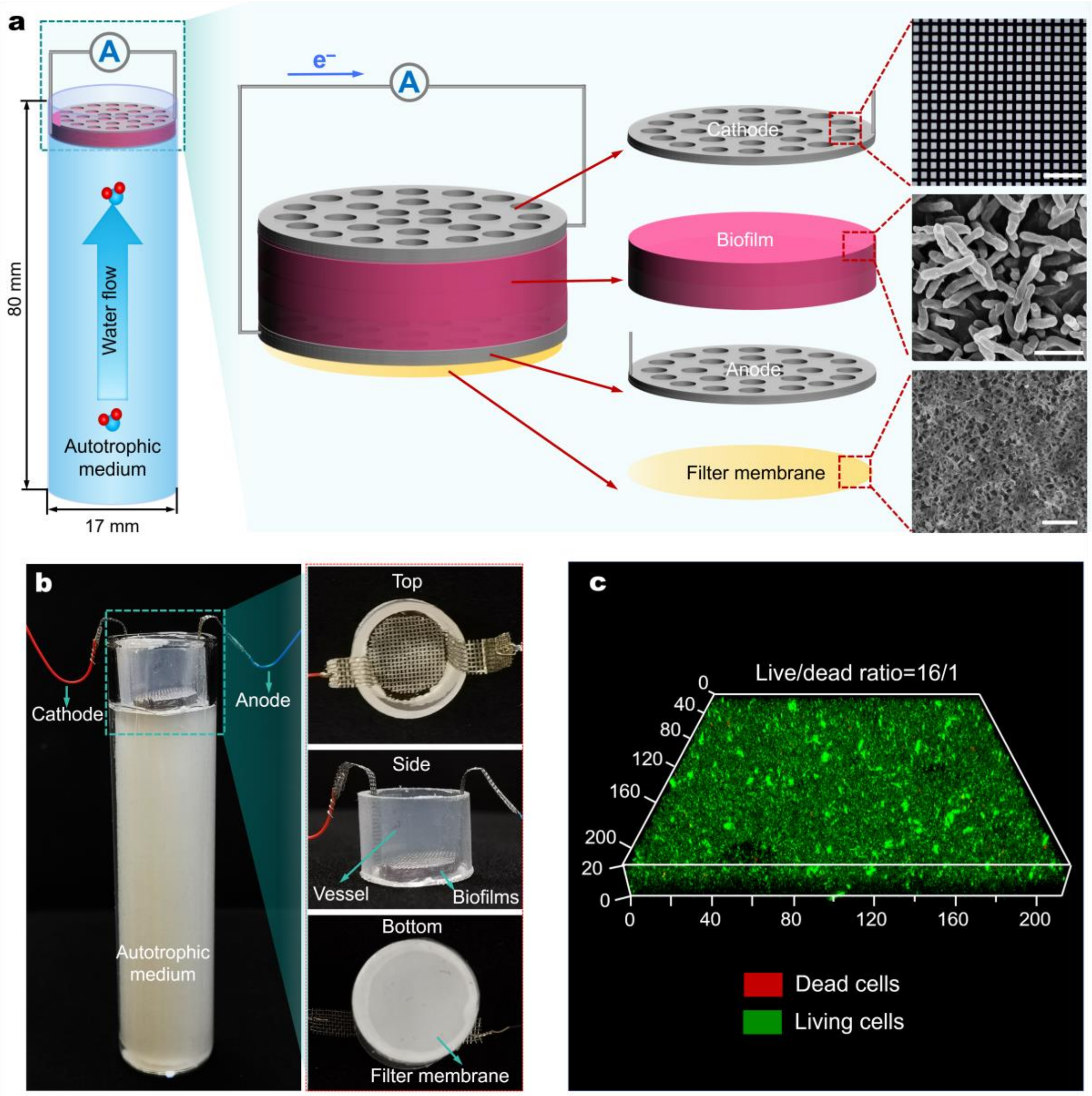

Supplementary Fig. 2 | Schematic and photographic illustrations of the microbial hydrovoltaic energy generation system (M-HEG). a, Schematic illustration of the M-HEG system. Optical microscopy image of the electrodes (top electrode and bottom electrode), scanning electron microscopy (SEM) images of the *R. palustris* biofilm and the filter membrane. **b**, Photographic illustration of the constructed M-HEG. **c**, Three-dimensional confocal laser scanning microscopy (CLSM) image of the *R. palustris*-based M-HEG biofilm at 0 day. The resulting fluorescence data was analyzed by a ZEN software and Image J software to obtain cell viability. The green and red dots represent the living cells and the dead cells, respectively, and the unit is micrometer (μm). Scale bars: 200 μm in **a** for top electrode, 2 μm in **a** for biofilms, 10 μm in **a** for filter membrane.

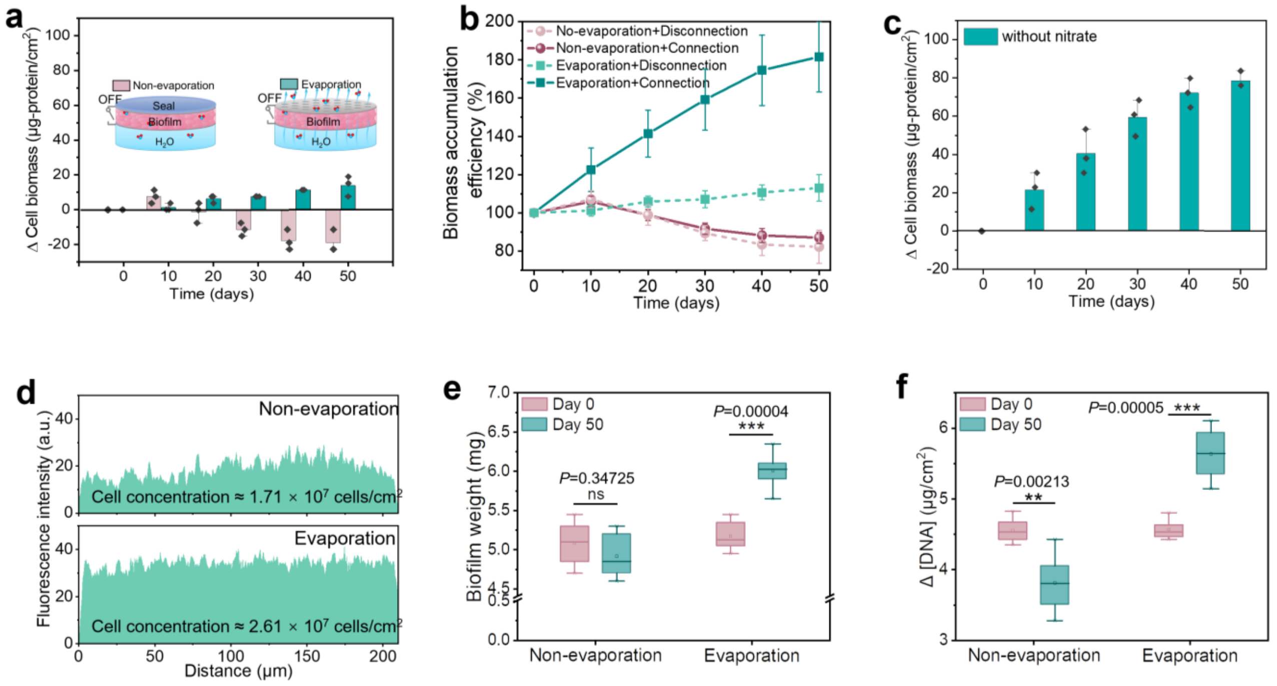

Supplementary Fig. 3 | Microbial growth powered by hydrovoltaic electrons. a, Cell biomass (total protein) accumulation in the absence of hydrovoltaic effect (non-evaporation with elctrical connection between top electrode and bottom electrode, and evaporation without elctrical connection). **b**, Biomass accumulation efficiency in the presence and absence of hydrovoltaic effect (three deletional controls). **c**, Cell biomass (total protein) accumulation of control experiment by removing nitrate in an autotrophic Medium II. **d**, Biomass profiles in biofilm in the presence and absence of hydrovoltaic effect, which were obtained from CLSM images, and analyzed by the Image J software to characterize the changes of biomass with thickness of biofilm[7]. Green area represents the biomass of biofilm. **e**, Biofilm weight in the presence and absence of hydrovoltaic effect. **f**, Total DNA concentration of biofilm in the presence and absence of hydrovoltaic effect. Data represent mean ± standard deviation (SD) from n = 3 technical replicates from one experiment. Statistical analysis was conducted with paired two-tailed t-tests: $^{**}P \leq 0.01$, $^{***}P \leq 0.001$, ns represents no significant difference.

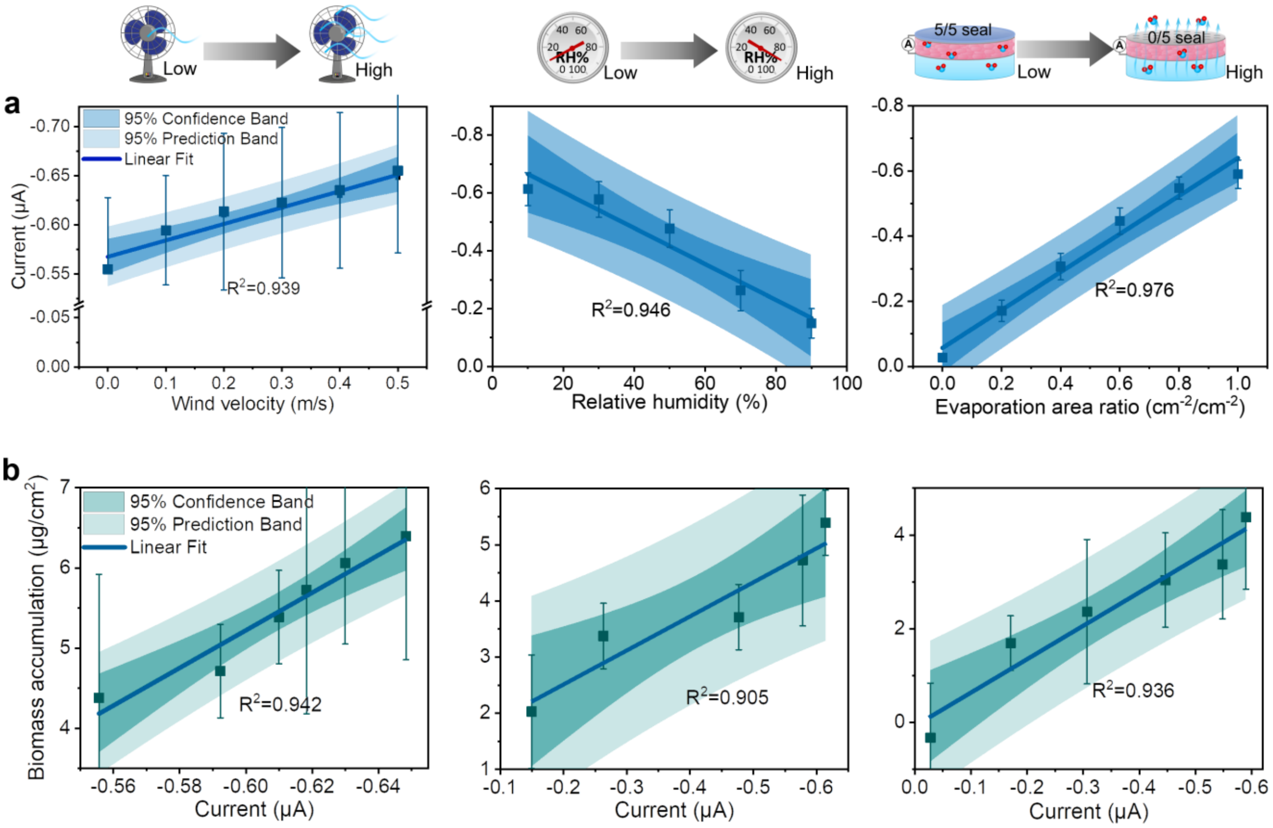

Supplementary Fig. 4 | Correlation analyses between water evaporation and microbial growth. a, Positive relationships between the WE-HE and the evaporation rate (wind velocity, relative humidity, and evaporation area ratio). **b**, Positive relationships between the microbial growth (biomass accumulation) and WE-HE (current intensity). The correlation analyses were conducted by the Origin 2018 software. Line connects the 95% confidence intervals of the points. Data represent mean ± SD from n = 3 technical replicates from one experiment. Statistical analysis was conducted with paired two-tailed t-tests.

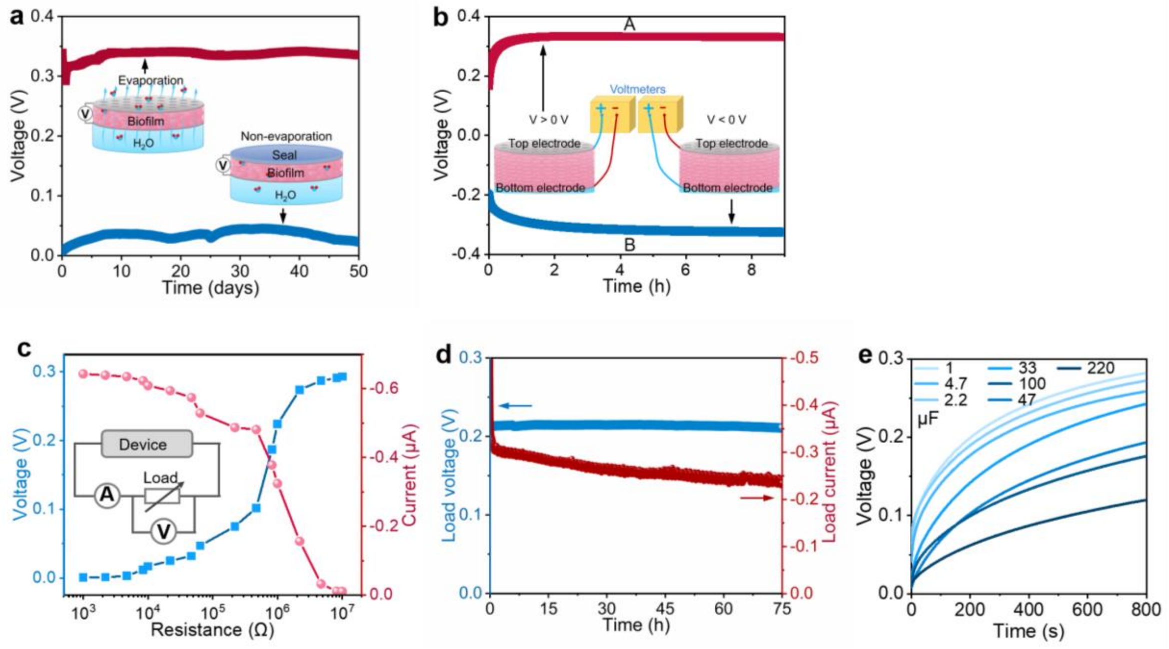

Supplementary Fig. 5 | Electric characterizations for hydrovoltaic electron generation in *R. palustris* biofilms. a, Open-circuit voltage generated by the biofilms through evaporation and non-evaporation of water. **b**, Switching-polarity test of a hydrovoltaic voltage. The induced voltage reverses its sign when the electric circuit connection of device is exchanged without changing the construction and position of device, which can be an indicator for true electricity generation[8]. For pattern A, the top electrode and bottom electrode are connected to the positive pole and negative pole of voltmeter, respectively, resulting in a voltage higher than 0 V. For pattern B, the top electrode and bottom electrode are connected to the negative pole and positive pole of voltmeter, respectively, resulting in a voltage lower than 0 V. Red and blue curves are the corresponding voltage of the device before and after it is turned over, respectively. **c**, Voltage and current changes with different load resistances for the M-HEG system, the inset shows a schematic diagram of the test circuit. **d**, Long-time load voltage and current with resistance of 1 MΩ. **e**, Voltage-time curves of commercial capacitors (1, 2.2, 4.7, 33, 47, 100 and 220 μF) charged by hydrovoltaic electrons.

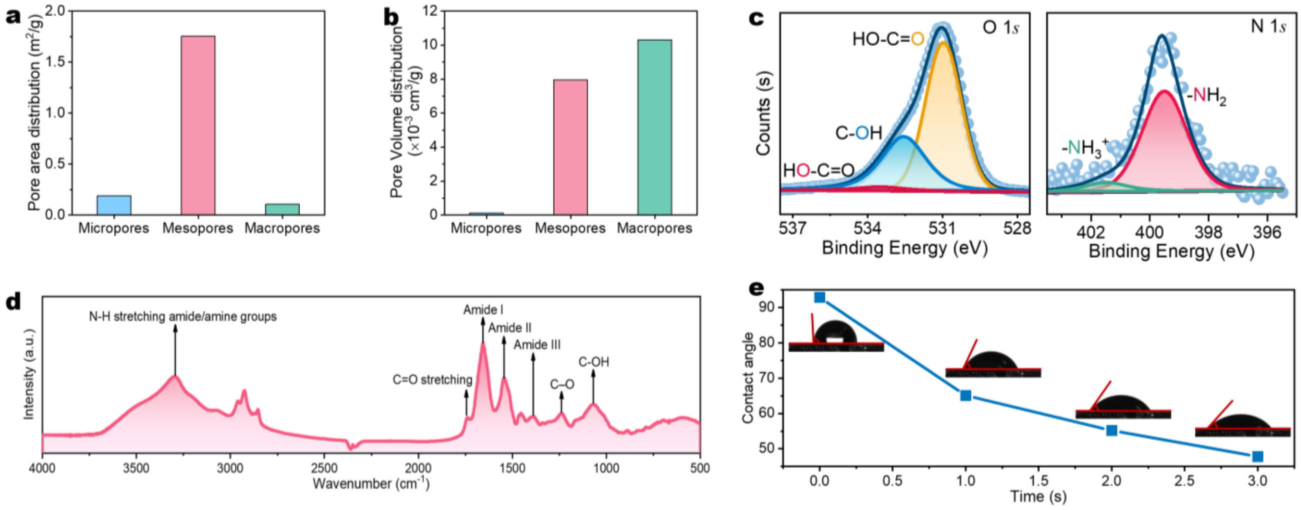

Supplementary Fig. 6 | Composition and structure characterizations of *R. palustris* biofilm. a, Pore area distribution of the *R. palustris* biofilm. **b**, Pore volume distribution of the *R. palustris* biofilm. **c**, X-ray photoelectron spectroscopy (XPS) images of oxygen, and nitrogen elements. **d**, Fourier transform infrared (FTIR) spectroscopy of the *R. palustris* biofilm. Hydrophilic groups are mainly from the abundant proteins and polysaccharides in *R. palustris* cells. **e**, Surface contact angle changes of the *R. palustris* biofilm within 3 seconds, the inset images show the contact angle of water droplets on a *R. palustris* biofilm.

The biofilms predominantly consisted of mesopores (2–50 nm, approximately 85.6%), with micropores (0–2 nm, 9.2%) and macropores (>50 nm, 5.2%) present in lower proportions[9]. The majority of the pore volume was attributed to macropores (56.0%) and mesopores (approximately 43.2%), with micropores contributing a minimal fraction (0.8%). The abundance of mesopores may enhance water permeation through the biofilm, thereby supporting capillary forces[10].

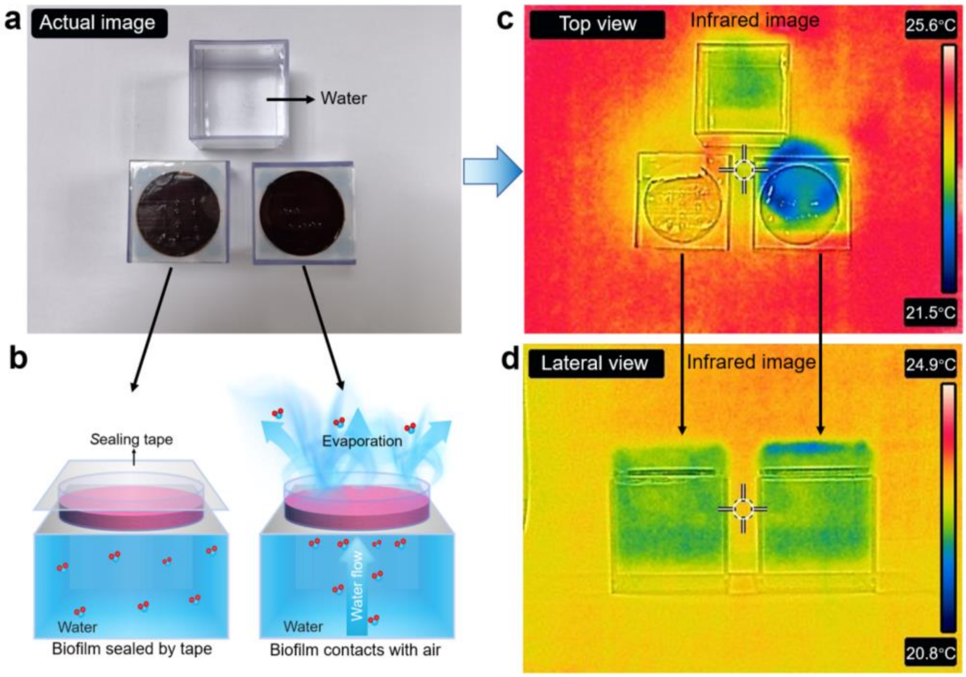

Supplementary Fig. 7 | Infrared thermal images of the *R. palustris* biofilms. a,**b**, Actual image (**a**) and the corresponding schematic diagram (**b**) of the biofilms. **c**, Top view of the infrared thermal image of the biofilm contacting with air, the biofilm sealed by tape and pure water without biofilm. **d**, Lateral view of the infrared thermal image of the biofilm contacting with air and the biofilm sealed by tape.

The water evaporation process is shown to significantly lower the biofilm's surface temperature compared to the non-evaporating biofilm, illustrating that the evaporation process facilitates the absorption of ambient energy, subsequently converting it into the phase transition energy required for the transition of water from the liquid to the gaseous state.

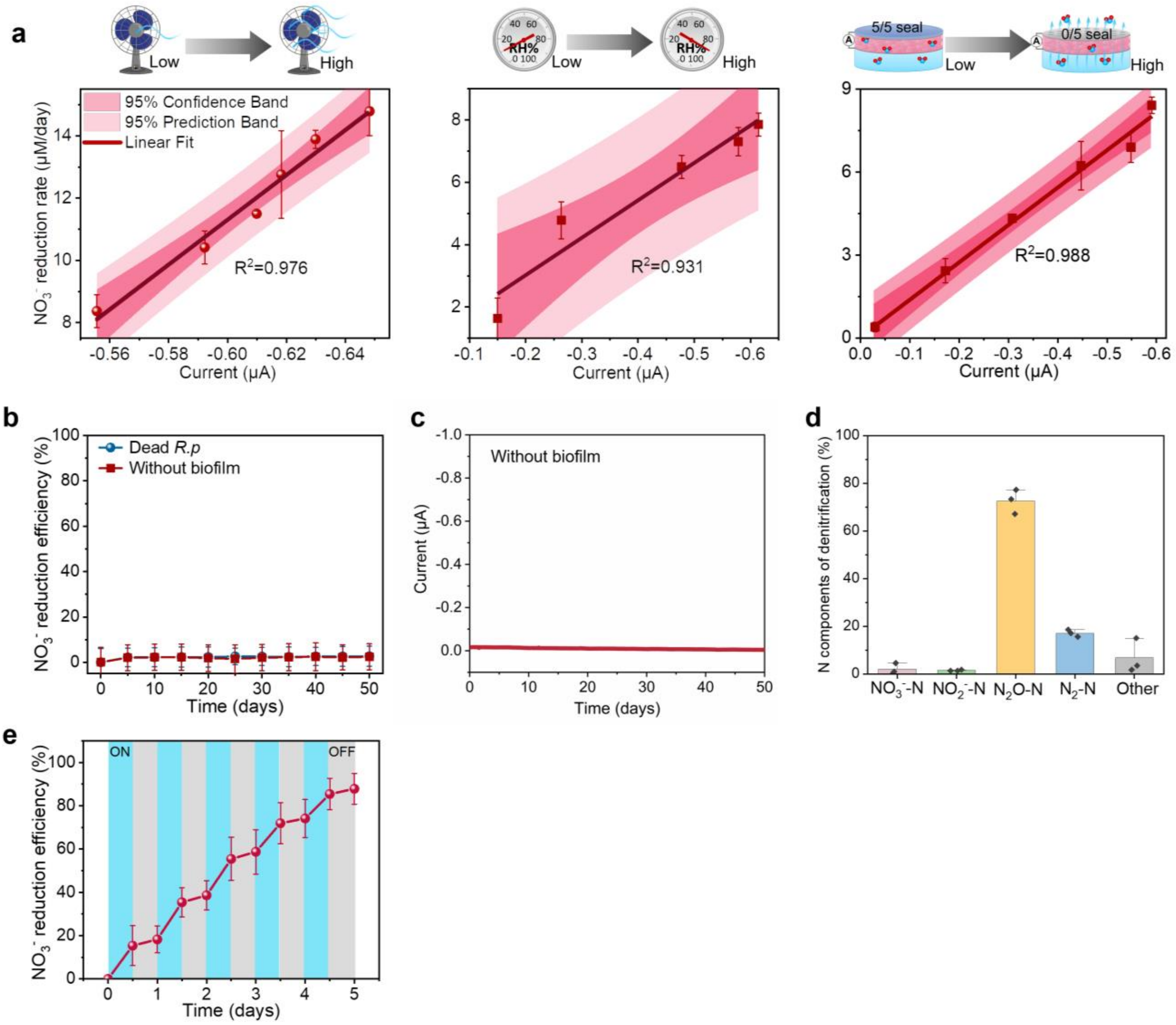

Supplementary Fig. 8 | NO_3^- reduction performance by the *R. palustris* biofilm. a, Positive relationships between the NO_3^- reduction and WE-HE (current intensity). The correlation analyses were conducted by the Origin 2018 software. Line connects the 95% confidence intervals of the points. **b**, Typical time course of NO_3^- reduction performance in the absence of hydrovoltaic effect (dead *R. palustris* biofilm and without *R. palustris* biofilm). **c**, Hydrovoltaic current of abiotic control (without *R. palustris* biofilm). **d**, Nitrogenous components of denitrification driven by hydrovoltaic electrons at day 50. **e**, NO_3^- reduction by the WE-HE under an interval water evaporation (ON-OFF) cycles. The blue and gray backgrounds indicate that the hydrovoltaic system with (ON) and without (OFF) water evaporation process. Data represent mean ± SD from n = 3 technical replicates from one experiment.

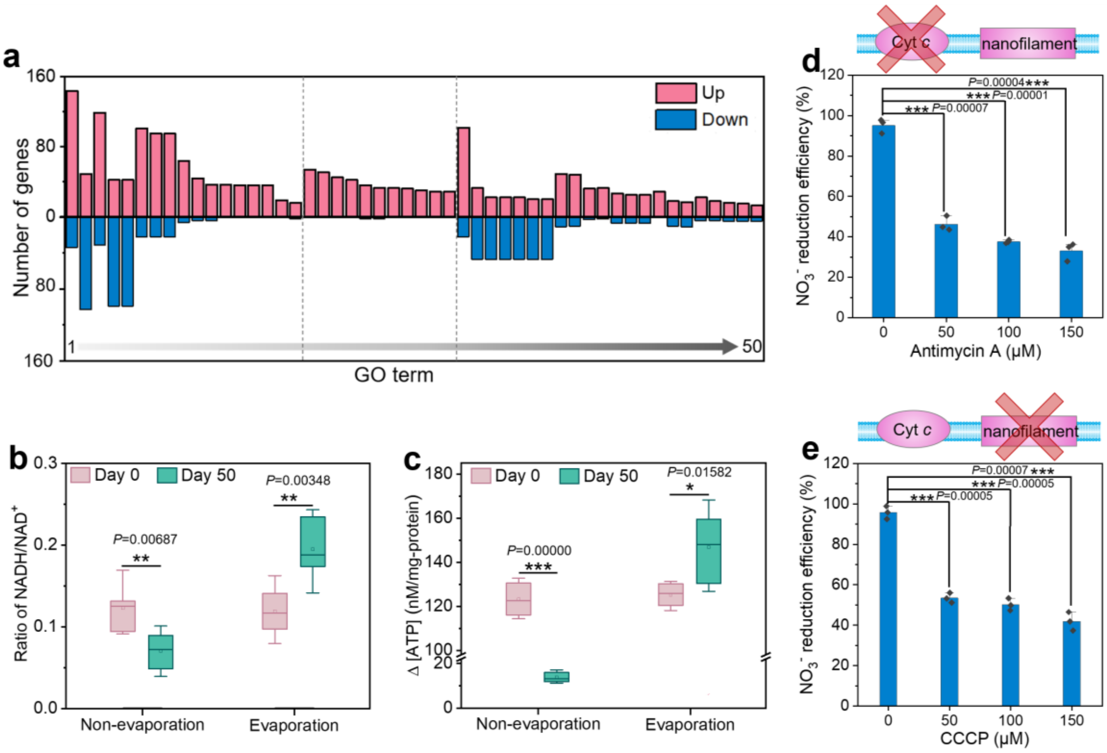

Supplementary Fig. 9 | Metabolism analysis for *R. palustris* biofilms. a, Top 50 genes with significant enrichment analyzed by gene ontology (GO) enrichment of differentially expressed genes: (1) Macromolecule metabolic process, (2) localization, (3) cellular macromolecule metabolic process, (4) establishment of localization, (5) transport, (6) gene expression, (7) macromolecule biosynthetic process, (8) cellular macromolecule biosynthetic process, (9) protein metabolic process, (10) cellular protein metabolic process, (11) cellular amide metabolic process, (12) amide biosynthetic process, (13) peptide metabolic process, (14) peptide biosynthetic process, (15) translation, (16) generation of precursor metabolites and energy, (17) cellular component organization, (18) intracellular, (19) intracellular part, (20) cytoplasm, (21) protein-containing complex, (22) organelle, (23) non-membrane-bounded organelle, (24) intracellular organelle, (25) cytoplasmic part, (26) intracellular non-membrane-bounded organelle, (27) ribonucleoprotein complex, (28) ribosome, (29) nucleic acid binding, (30) transporter activity, (31) hydrolase activity, acting on acid anhydrides, in phosphorus-containing anhydrides, (32) hydrolase activity, acting on acid anhydrides, (33) pyrophosphatase activity, (34) nucleoside-triphosphatase activity, (35) ATPase activity, (36) cation binding, (37) metal ion binding, (38) electron transfer activity, (39) structural molecule activity, (40) transition metal ion binding, (41) metal cluster binding, (42) iron-sulfur cluster binding, (43) structural constituent of ribosome, (44) ion transmembrane transporter activity, (45) inorganic molecular entity transmembrane transporter activity, (46) tetrapyrrole binding, (47) heme binding, (48) cation transmembrane transporter activity, (49) inorganic cation transmembrane transporter activity, (50) monovalent inorganic cation transmembrane transporter activity. **b**, $NADH/NAD^+$ ratio. **c**, Adenosine triphosphatase (ATP) concentration. **d**,**e**, NO_3^- reduction efficiency after the addition of different inhibitors of electron transfer chains. Antimycin A is an inhibitor of cytochrome bc_1, and carbonyl cyanide m-chlorophenyl hydrazine (CCCP) is an inhibitor of the formation of nanofilaments via the dissipation of proton motive force[11,12]. Cyt *c* represents C-type cytochromes, nanofilament represents protein nanowires and flagella. The red "×" symbol represents an addition of inhibitor. Data represent mean ± SD from $n = 3$ technical replicates from one experiment. Statistical analysis was conducted with paired two-tailed *t*-tests: $*P \leq 0.05$, $**P \leq 0.01$, $***P \leq 0.001$.

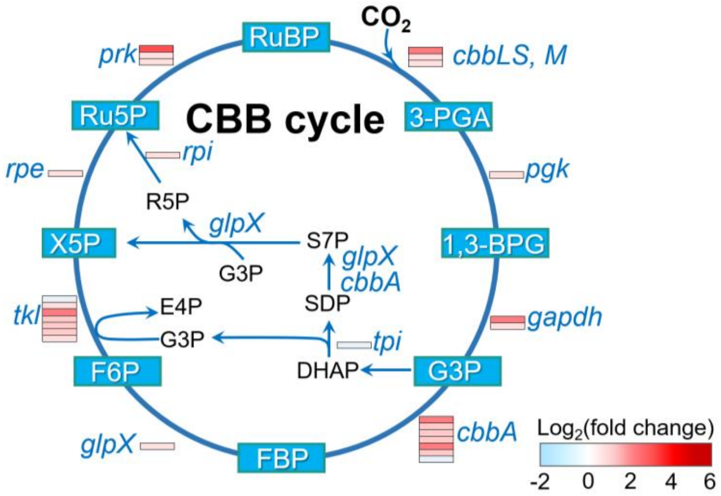

Supplementary Fig. 10 | Pathway analysis of the Calvin-Benson-Bassham (CBB) cycle in *R. palustris*. Differential expression analysis of genes encoding CBB cycle. RuBP, ribulose 1,5-bisphosphate; 3-PGA, 3-phosphoglycerate; 1,3-BPG, 1,3-bisphosphoglycerate; G3P, glyceraldehyde 3-phosphate; FBP, fructose 1,6-bisphosphate; F6P, fructose 6-phosphate; X5P, xylulose 5-phosphate; Ru5P, ribulose 5-phosphate; R5P, ribose 5-phosphate; E4P, erythrose 4-phosphate; S7P, sedoheptulose-7-phosphate; DHAP, dihydroxy-acetone-phosphate; SDP, sedoheptulose-1,7-bisphosphate; *cbbLS* and *cbbM*, form I and form II ribulose-1,5-bisphosphate carboxylase/oxygenase, respectively; *pgk*, phosphoglycerate kinase; *gapdh*, glyceraldehyde 3-phosphate dehydrogenase; *cbbA*, fructose 1,6-bisphosphate aldolase; *glpX*, fructose-1,6-bisphosphatase 1 and 2; *tkl*, transketolase; *rpe*, ribulose-phosphate 3-epimerase; *prk*, phosphoribulokinase; *rpi*, ribose-5-phosphate isomerase; *tpi*, triose-3-phosphate isomerase[11]. Data represent mean ± SD from $n = 3$ technical replicates from one experiment. Statistical analysis was conducted with paired two-tailed *t*-tests: $P < 0.05$. All P values are provided in Source Data.

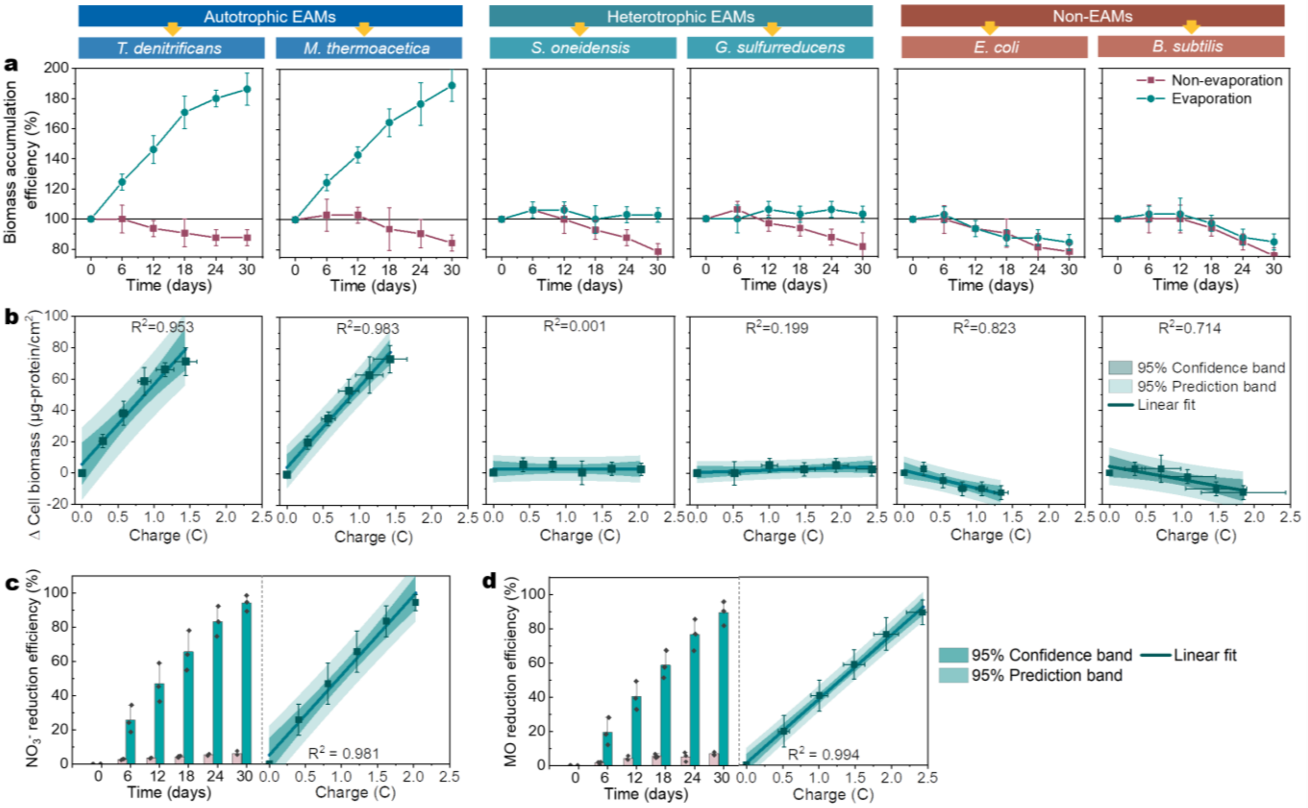

Supplementary Fig. 11 | Hydrovoltaic energy utilized by other microorganisms. a, Biomass accumulation efficiency with different microorganisms (*T. denitrificans*, *M. thermoacetica*, *S. oneidensis*, *G. sulfurreducens*, *E. coli* and *B. subtilis*) by hydrovoltaic system with and without water evaporation. **b**, Positive relationships between cell biomass accumulation and generated charge quantity (hydrovoltaic electrons) by natural water evaporation in hydrovoltaic system. **c**, Typical time course of NO_3^- reduction in the hydrovoltaic system with and without water evaporation, and positive relationships between NO_3^- reduction efficiency and generated charge quantity (hydrovoltaic electrons) by natural water evaporation. **d**, Typical time course of methyl orange (MO) reduction in the hydrovoltaic system, and positive relationships between MO reduction efficiency and generated charge quantity (hydrovoltaic electrons) by natural water evaporation. Data represent mean ± SD from *n* = 3 technical replicates from one experiment.

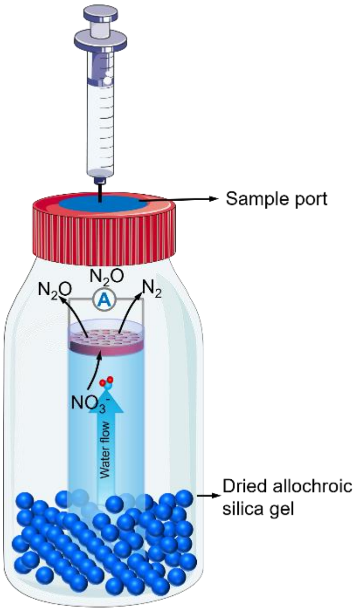

Supplementary Fig. 12 | Device diagram of a small-scale sealed reactor for measurement of the gas products (N_2 and N_2O) produced during the denitrification process driven by hydrovoltaic electrons.

Supplementary References

1. Liu, X. et al. Power generation from ambient humidity using protein nanowires. *Nature* **578**, 550-554 (2020).
2. Liu, X. et al. Microbial biofilms for electricity generation from water evaporation and power to wearables. *Nat. Commun.* **13**, 4369 (2022).
3. Fang, S. et al. Turning dead leaves into an active multifunctional material as evaporator, photocatalyst, and bioplastic. *Nat. Commun.* **14**, 1203 (2023).
4. Ye, J. et al. A facile and fast strategy for cathodic electroactive-biofilm assembly via magnetic nanoparticle bioconjugation. *Biosens. Bioelectron.* **190**, 113464 (2021).
5. Huang, S. et al. Sunlight significantly enhances soil denitrification via an interfacial biophotoelectrochemical pathway. *Environ. Sci. Technol.* **57**, 7733-7742 (2023).
6. Heidrich, E., Curtis, T. & Dolfing, J. Determination of the internal chemical energy of wastewater. *Environ. Sci. Technol.* **45**, 827-832 (2011).
7. Hu, A. et al. Metal-free semiconductor-based bio-nano hybrids for sustainable CO_2-to-CH_4 conversion with high quantum yield. *Angew. Chem.* **134**, e202206508 (2022).
8. Xue, G. et al. Water-evaporation-induced electricity with nanostructured carbon materials. *Nat. Nanotechnol.* **12**, 317-321 (2017).
9. Ren, G. et al. Hydrovoltaic effect of microbial films enables highly efficient and sustainable electricity generation from ambient humidity. *Chem. Eng. J.* **441**, 135921 (2022).
10. Hu, Q. et al. Water evaporation–induced electricity with *Geobacter sulfurreducens* biofilms. *Sci. Adv.* **8**, eabm8047 (2022).
11. Guzman, M. S. et al. Phototrophic extracellular electron uptake is linked to carbon dioxide fixation in the bacterium *Rhodopseudomonas palustris*. *Nat. Commun.* **10**, 1355 (2019).
12. Minamino, T. & Namba, K. Distinct roles of the FliI ATPase and proton motive force in bacterial flagellar protein export. *Nat. Biotechnol.* **451**, 485-488 (2008).
