# Supplementary material for: Growth of electroautotrophic microorganisms using hydrovoltaic energy through natural water evaporation
Source: Nat Commun. 2024 Jun 11;15:4992. doi: 10.1038/s41467-024-49429-0 (PMC11166942; doi:10.1038/s41467-024-49429-0)
Supplement: Supplementary file 3 — Description of Additional Supplementary Files [file 41467_2024_49429_MOESM3_ESM.pdf]

Description of Additional Supplementary Files:

Supplementary Movie 1: R. palustris cells still exhibited good motility after 50 days of water evaporation.
